# *MARVELD2* (*DFNB49*) Mutations in the Hearing Impaired Central European Roma Population - Prevalence, Clinical Impact and the Common Origin

**DOI:** 10.1371/journal.pone.0124232

**Published:** 2015-04-17

**Authors:** Ivica Mašindová, Andrea Šoltýsová, Lukáš Varga, Petra Mátyás, Andrej Ficek, Miloslava Hučková, Martina Sůrová, Dana Šafka-Brožková, Saima Anwar, Judit Bene, Slavomír Straka, Ingrid Janicsek, Zubair M. Ahmed, Pavel Seeman, Béla Melegh, Milan Profant, Iwar Klimeš, Saima Riazuddin, Ľudevít Kádasi, Daniela Gašperíková

**Affiliations:** 1 Laboratory of Diabetes and Metabolic Disorders & DIABGENE, Institute of Experimental Endocrinology, Slovak Academy of Sciences, Bratislava, Slovakia; 2 Department of Molecular Biology, Faculty of Natural Sciences, Comenius University, Bratislava, Slovakia; 3 Institute of Molecular Physiology and Genetics, Slovak Academy of Sciences, Bratislava, Slovakia; 4 Department of Otorhinolaryngology—Head and Neck Surgery, Faculty of Medicine and University Hospital, Comenius University, Bratislava, Slovakia; 5 Department of Medical Genetics, University of Pécs, Clinical Centre, Pécs, Hungary; 6 Center for Molecular Medicine, Slovak Academy of Sciences, Bratislava, Slovakia; 7 DNA Laboratory, Department of Paediatric Neurology, Charles University 2nd Medical School and University Hospital Motol, Prague, Czech Republic; 8 Department of Otorhinolaryngology Head & Neck Surgery, School of Medicine, University of Maryland, Baltimore, Maryland, United States of America; 9 Szentagothai Research Centre, University of Pécs, Pécs, Hungary; 10 Department of Otorhinolaryngology—Head and Neck Surgery, Faculty Hospital of J. A. Reiman, Prešov, Slovakia; Hadassah-Hebrew University Medical Center, ISRAEL

## Abstract

**Background:**

In the present study we aimed: 1) To establish the prevalence and clinical impact of *DFNB49* mutations in deaf Roma from 2 Central European countries (Slovakia and Hungary), and 2) to analyze a possible common origin of the c.1331+2T>C mutation among Roma and Pakistani mutation carriers identified in the present and previous studies.

**Methods:**

We sequenced 6 exons of the *MARVELD2* gene in a group of 143 unrelated hearing impaired Slovak Roma patients. Simultaneously, we used RFLP to detect the c.1331+2T>C mutation in 85 Hungarian deaf Roma patients, control groups of 702 normal hearing Romanies from both countries and 375 hearing impaired Slovak Caucasians. We analyzed the haplotype using 21 SNPs spanning a 5.34Mb around the mutation c.1331+2T>C.

**Results:**

One pathogenic mutation (c.1331+2T>C) was identified in 12 homozygous hearing impaired Roma patients. Allele frequency of this mutation was higher in Hungarian (10%) than in Slovak (3.85%) Roma patients. The identified common haplotype in Roma patients was defined by 18 SNP markers (3.89 Mb). Fourteen common SNPs were also shared among Pakistani and Roma homozygotes. Biallelic mutation carriers suffered from prelingual bilateral moderate to profound sensorineural hearing loss.

**Conclusions:**

We demonstrate different frequencies of the c.1331+2T>C mutation in hearing impaired Romanies from 3 Central European countries. In addition, our results provide support for the hypothesis of a possible common ancestor of the Slovak, Hungarian and Czech Roma as well as Pakistani deaf patients. Testing for the c.1331+2T>C mutation may be recommended in *GJB2* negative Roma cases with early-onset sensorineural hearing loss.

## Introduction

Hearing impairment is one of the most common birth defects in humans. Approximately 50% of all congenital deafness cases are attributable to genetic causes [1]. To date, nonsyndromic sensorineural hearing loss (SNHL) is known to be associated with 30 genes with autosomal dominant inheritance and more than 50 genes with autosomal recessive inheritance [2]. The *MARVELD2* gene (MAL and Related proteins for Vesicle trafficking and membrane Link Domain containing 2), also known as *TRIC* and included in the autosomal recessive group, is located on the chromosome 5q13.2 and linked to the *DFNB49* locus [3, 4]. Human *MARVELD2* gene encodes the MARVELD2/tricellulin protein composed of 558 amino acids [4]. This transmembrane protein is mainly concentrated in tricellular tight junctions (tTJ) in the epithelial cells of all tissues, including cochlear supporting cells, hair cells and marginal cells of stria vascularis, and also weakly present in bicellular tight junctions (bTJ) [5, 6]. In the tTJ, tricellulin provides connection between three epithelial cells, thus playing a critical role in forming an epithelial barrier against paracellular flux of ions and solutes, which is essential to maintain the ion composition of inner ear fluids and proper hearing function [5]. In humans, mutations in the *MARVELD2* gene lead to nonsyndromic, bilateral, prelingual moderate to profound deafness [4, 7]. However, the *Marveld2* knock-in mice (ortholog mutation to human c.1498C>T, p.Arg500*) had progressive hair cell degeneration accompanied by rapidly progressing hearing loss, although the endocochlear potential and function of *stria vascularis* were unaffected. Ultrastructural changes of TJ’s in the sensory epithelium of the inner ear may selectively change the paracellular permeability of ions or small molecules, resulting in a toxic microenvironment for cochlear hair cells [8].

The nonsyndromic autosomal recessive deafness locus *DFNB49* was initially mapped by Ramzan et al. (2005) in two Pakistani families. To date, six deafness causing recessive mutations in this gene have been identified in 15 families worldwide [4, 7, 9, 10]. Initially, these mutations were only found in patients of Pakistani origin [4, 7]. But more recently, the mutation c.1331+2 T*>*C (IVS4+2T*>*C) has also been identified in the European Roma population, possibly contributing significantly to the deafness etiology within this ethnic group [10]. Mutations in the *MARVELD2* gene may therefore represent an important cause of nonsyndromic hearing loss in both of these populations with a possible common ancestry.

The Romanies are a unique population that according to many relevant historical studies migrated from South Asia into Europe between the 5^th^ and 10^th^ century [11, 12]. Genome-wide studies estimate that the initial founder Roma population originated from Northwest India approximately 1.5 thousand years ago. They migrated through the Middle East and spread into Europe about 850–900 years ago [12, 13]. Here, the Roma population forms a conglomerate of genetically isolated founder subpopulations [14], characterized by high inbreeding rate [15] and increased frequency of multiple autosomal recessive disorders compared to the majority Caucasian population.

Studies on several hereditary disorders occurring in Roma populations from Central Europe have been performed, including congenital glaucoma [16–18], phenylketonuria [19], hereditary thrombophilia [20] and hemochromatosis [21] in Slovakia; pontocerebellar hypoplasia type 1 [22] and Charcot-Marie-Tooth disease type 4 [23] in the Czech Republic; carnitine-responsive cardiomyopathy [24] and galactokinase deficiency [25] in Hungary. The most frequent genetic cause of hearing loss identified so far in the Slovak Roma population is the c.71G>A (p.Trp24*) mutation in *GJB2* gene [26]. This corresponds to data from several other European countries [27–29].

In our present study, the previously detected prevalence of the *MARVELD2* mutation in Czech Roma patients is taken into account with the above mentioned unique geographic and demographic history of the Roma population in Europe, and with respect to the common geopolitical history shared by three neighboring countries (Slovakia and Czech Republic in former Czechoslovakia twenty years ago or Slovakia, Czech Republic and Hungary in Austro-Hungarian Empire one hundred years ago). Thus, we sought to determine: 1) The prevalence of *MARVELD2* mutations in the Slovak and Hungarian hearing impaired Roma patients, 2) whether mutations found in the Roma population of the three Central European countries have a common origin, and 3) evaluate a possible linkage to the presumed common ancestor with the Pakistani population.

## Patients and Methods

### Study source population and inclusion criteria

#### Slovakia

One hundred forty-three unrelated hearing impaired Roma individuals without biallelic *GJB2/GJB6* mutations were selected for *MARVELD2* analysis. Of these, DNA samples from 86 patients came from DIABGENE Laboratory, IEE SAS. They were collected at boarding schools for hard of hearing located throughout Slovakia (Bratislava, Kremnica, Lučenec, Krásnohorské Podhradie, Levoča, Prešov) and at the ORL department of the University Hospital in Bratislava in the frame of nation-wide screening for hereditary hearing loss in Slovakia (2010–2013). Another 57 samples originated from Dept. of Molecular Biology, Faculty of Natural Sciences, Bratislava and were selected from a DNA repository containing samples from previous studies on nonsyndromic deafness and samples sent by clinical geneticists.

A control group of 200 unrelated normal hearing Roma individuals was selected and tested for presence of the c.1331+2T>C mutation to assess its frequency in the general Roma population.

To screen for the c.1331+2T>C mutation in Slovak patients of non-Roma origin, a control group of 375 *GJB2* negative unrelated Slovak (Caucasian) patients, matched for the hearing loss phenotype (nonsyndromic severe to profound bilateral sensorineural hearing loss of prelingual onset), was also tested.

All participants provided their written informed consent to participate in this study. This study has been approved by the Ethics Committee of the University Hospital in Bratislava, Slovakia.

#### Hungary

Eighty-five unrelated hearing impaired Roma patients, irrespective of their *GJB2* status, were analyzed for the presence of the identified c.1331+2T>C mutation in *MARVELD2* gene. They originated from Northeastern Hungary (Borsod-Abaúj-Zemplén County), near the Slovakian border.

The control group composed of 502 unrelated normal hearing Roma individuals was used to estimate the mutation frequency in the Hungarian Roma population. Approximately half of the controls also came from the Northeast Borsod-Abaúj-Zemplén County, and the other half from the Baranya County in the southern part of Hungary.

#### Czech Republic

Five *MARVELD2*, c.1331+2T>C homozygous DNA samples from 3 families, identified in a previous study [10], were genotyped for common haplotype determination analysis.

#### Pakistan

Four homozygous patients with *MARVELD2* related deafness from two families, one family harbouring the c.1331+2T>C mutation and the other with the c.1183-1G>A mutation, were analyzed for a common haplotype. Family PKDF800 was enrolled from the Punjab province, while family PKDF941 was originated from the Khyber Pakhtunkhwa province of Pakistan. All patients were analyzed for a common haplotype.

### *MARVELD2* gene sequencing and detection of the c.1331+2T>C mutation

Direct sequencing of the six exons and intron-exon boundaries of the *MARVELD2* gene was performed using previously published primers [10]. PCR products were sequenced according to standard procedures (Big Dye Terminator Sequencing Kit v3.1, Life Technologies, USA) and analyzed on the ABI 3500 genetic analyzer (Life Technologies, USA). Sequences were compared with reference sequence from GenBank NG_017201.1 using the SeqScape software (version 2.7; Life Technologies, USA).

The identified c.1331+2T>C mutation was subsequently screened in population samples by restriction digest detection after PCR amplification. DNA samples were amplified using 5´CCC ACC TGA TCT TCT TCC TC 3´ (forward) and 5´AAA GCC AGA TTT TAT TCA TCC TCT A 3´ (reverse) primers and digested with Bsh1236I (BstUI) (Thermo Fisher Scientific, USA) to yield fragments of 727bp for the wild type allele and 480bp and 247bp for the mutant allele.

### *In silico* analysis of the novel variant

PolyPhen2 (http://genetics.bwh.harvard.edu/pph2/index.shtml) [accessed 20 April 2014], SIFT (http://sift.jcvi.org) [accessed 20 April 2014] and MutationTaster (mutationtaster.org) [accessed 20 April 2014] software were used to assess the potential pathogenicity of the novel variant.

### Haplotype analysis

Twenty one single nucleotide polymorphisms (rs542778, rs4699896, rs4976108, rs67911569, rs10059317, rs56103849, rs4252228, rs1168405, rs1168402, rs299086, rs299093, rs2434507, rs299075, rs299078, rs28652974, rs28409706, rs468467, rs188123810, rs467880, rs466930, and rs2133729) spanning 5.34 megabases around the c.1331+2T>C mutation were genotyped. Polymorphisms were selected from the dbSNP database with respect to their chromosomal position and minor allele frequency (MAF) value. Haplotype analysis was performed in seventeen c.1331+2T>C homozygous patients: 5 Slovak probands, 7 Hungarian probands, 5 Czech (3 probands, 2 relatives) and 4 Pakistani patients from 2 families (2 relatives homozygous for c.1331+2T>C mutation and 2 relatives with homozygous mutation c.1183-1G>A).

A control group of 20 unrelated hearing impaired Roma patients and 36 unrelated normal hearing Roma individuals without the c.1331+2T>C mutation was genotyped along to determine genetic variability of the selected SNP markers. All markers were genotyped by Sanger sequencing using ABI BigDye v3.1 chemistry after standard PCR amplification (5 PRIME master mix; 5 PRIME, D) and ExoSAP (Affymetrix, USA) purification.

### Audiological evaluation

Hearing thresholds were recorded by pure tone audiometry (PTA) or auditory steady state responses (ASSR), depending on the subject’s age and cooperation.

## Results

### DNA analysis of the *MARVELD2* gene in Slovak patients

Screening of 143 unrelated hearing impaired Roma individuals from Slovakia detected the c.1331+2T>C mutation in a homozygous state in five (3.5%) and in a heterozygous state in one affected individual. Subsequent sequencing of *MARVELD2* did not reveal any other pathogenic mutations. Additionally, five polymorphisms (c.98C>T, c.898T>A, c.1146+105G>A, c.1147-9T>G, c.1331+42G>A) were identified in homozygous state in five individuals and one variant of unknown pathogenicity (c.950G>A) (rs148416461) was found in heterozygous form. Bioinformatic tools predicted the c.950G>A variant to be probably pathogenic (PolyPhen2 score = 1.000; SIFT score = 0; MutationTaster score = 43).

Screening of 200 unrelated normal hearing Romanies revealed nine individuals heterozygous for the c.1331+2T>C mutation. However, this mutation was not found in the group of 375 hearing impaired non-Roma (Caucasian) patients.

### DNA analysis of the *MARVELD2* gene in Hungarian Roma patients

Analysis of the c.1331+2T>C mutation in the group of 85 unrelated deaf Hungarian Romanies identified seven homozygous and three heterozygous patients. In this group, the prevalence of c.1331+2T>C homozygotes was 8.23%. In the control group of 502 normal hearing Hungarian Romanies, the c.1331+2T>C mutation was found in 5 heterozygous individuals.

### Frequency of the c.1331+2T>C mutation

The observed frequency of the c.1331+2T>C allele in Slovak Roma hearing impaired patients was 3.85% (95% CI = 1.6–6.1%), while in unaffected Slovak Roma population the frequency was 2.25% (95% CI = 0.8–3.7%). The allele frequency of the mutation in the sample of Hungarian hearing impaired Roma patients was 10.0% (95% CI = 5.5–14.5%), which is nearly three times as much as in the Slovak deaf study group. On the other hand, the 0.5% (95% CI = 0.45–1.70%) allele frequency in the Hungarian healthy Roma population was approximately four times lower than in Slovak healthy Romanies. In the sample of 375 non-Roma Slovak patients with hearing loss, the c.1331+2T>C mutation was not detected.

### Haplotype analysis

**Fig 1:**
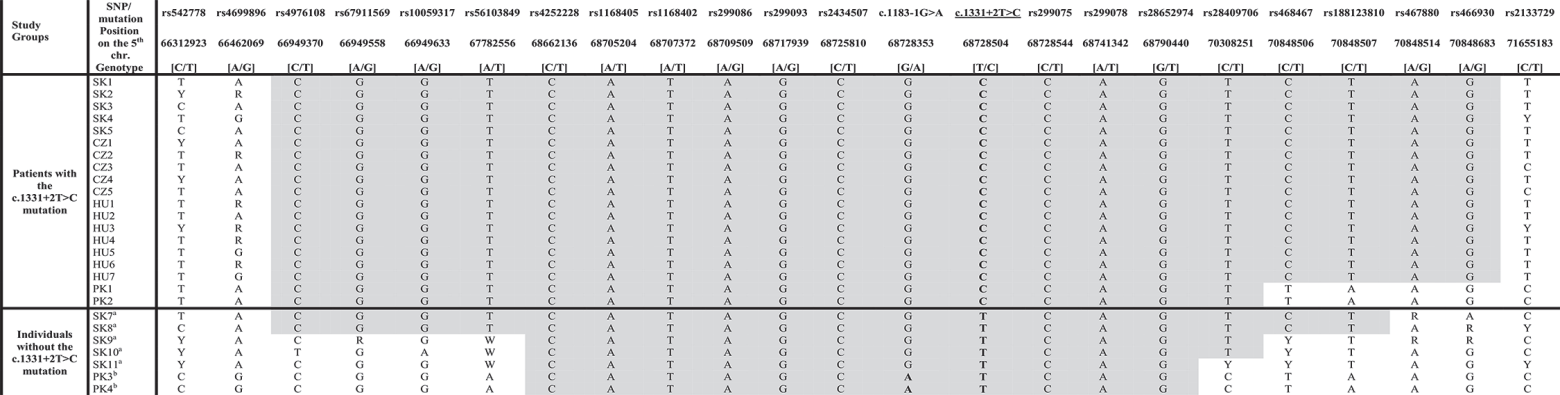
Common ancestral haplotypes of Slovak, Hungarian and Czech Romanies, and Pakistani patients. The haplotypes in Slovak (SK), Czech (CZ), Hungarian (HU) Roma and Pakistani patients with the c.1331+2T>C mutation, and haplotypes in Slovak Roma controls without the c.1331+2T>C mutation. The c.1331+2T>C mutation is shown in bold. The common haplotype is highlighted in light grey.^a^The most similar haplotypes found in 5 out of 56 control individuals. In the remaining 51 control individuals, haplotypes were significantly distinguishable.^b^The haplotype in Pakistani patients with c.1183-1G>A mutation.

**Fig 2:**
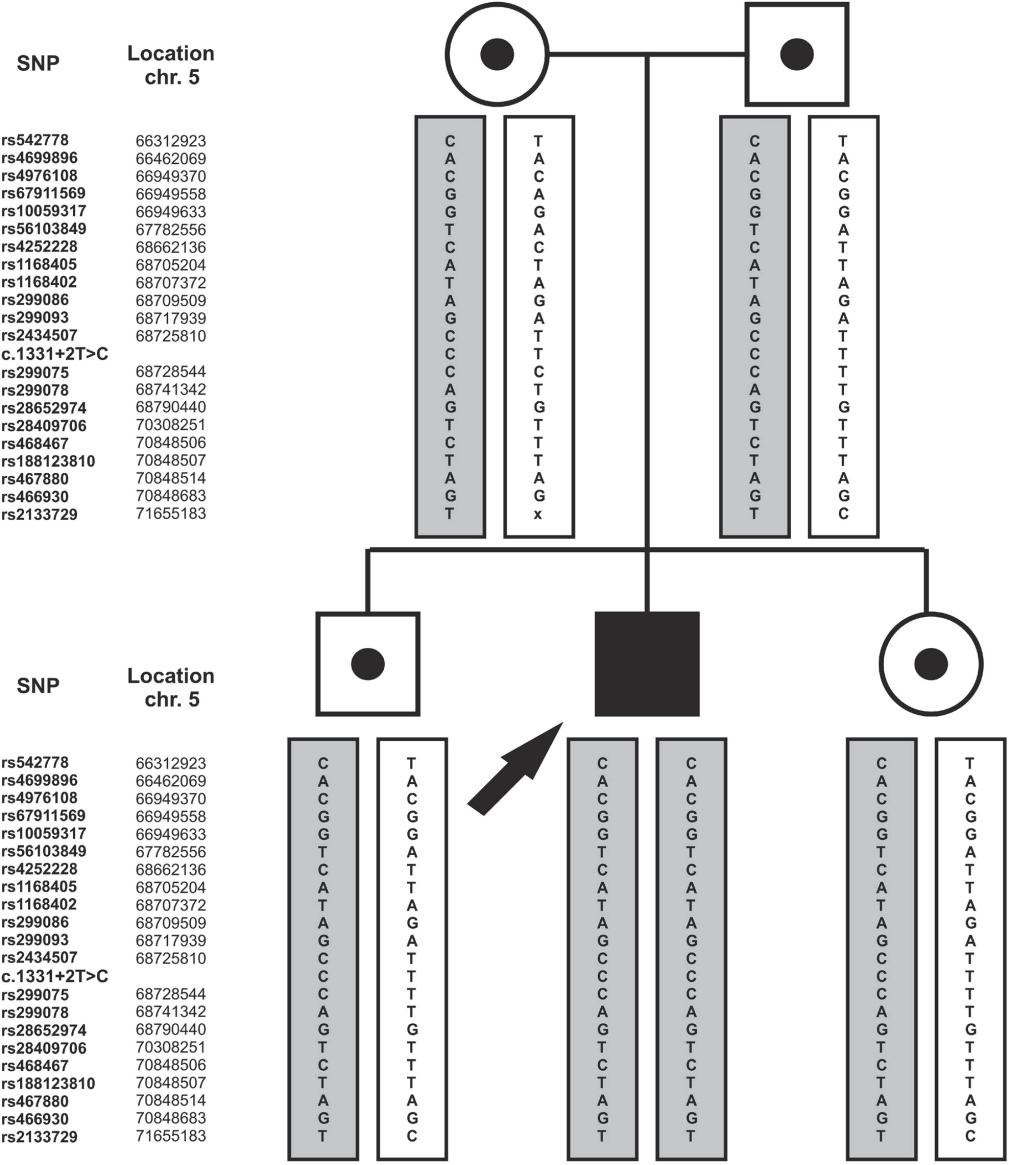
Cosegregation of haplotypes in a Slovak Roma family (SK5) with DFNB49 related deafness caused by the c.1331+2T>C mutation. The filled symbol represents the proband with hearing impairment; symbols with dot represent normal hearing carriers. Alleles forming the risk haplotype are shown in grey boxes.

To confirm the common ancestry of the c.1331+2T>C mutation in all patients from our study, as well as among the Pakistani and Czech subjects where the mutation was first detected, we analyzed 21 SNPs located within approximately 5.34 megabase region around the mutation. The identified common haplotype defined by 18 SNP markers (approx. 3.89 megabases), was shared by all Czech, Slovak and Hungarian Roma patients in a homozygous state, suggesting a common ancestor for this mutation in Central European Roma patients (Fig 1). To determine presence of the identified haplotype in the population sample, a selected subset of SNPs was also genotyped in 56 unrelated Roma individuals without the mutation. The haplotype spanning 18 SNPs was not detected in a homozygous state in any of the analyzed control samples, supporting the hypothesis of a common ancestry of this mutation among these patients. However, in five of control samples (showed in Fig 1), a shorter haplotype in homozygous state was detected. The rest of genotyped control samples did not possess homozygous haplotypes similar to the identified disease haplotype. To further support the existence of the common risk haplotype, the cosegregation of the identified haplotype with the c.1331+2T>C mutation was analyzed within a single family. As shown in the Fig 2, alleles forming the risk haplotypes are present in the affected and carrier individuals.

Fourteen common SNPs shared among two members of one Pakistani family and all Roma patients carrying the homozygous c.1331+2T>C mutation were found. In contrast, members of the second family of Pakistani origin harboring the c.1183-1G>A mutation possessed an identical homozygous region with the both the Pakistani and Roma patients of only nine SNPs wide (Fig 1).

### Hearing loss phenotype in the homozygotes

**Fig 3:**
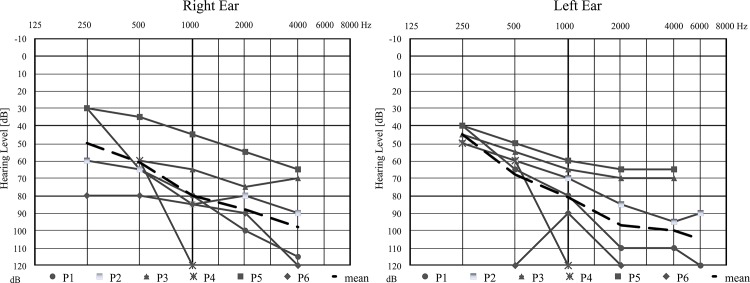
Hearing thresholds recorded by PTA in Slovak homozygous subjects. P1, P2, P3, P5, P6—positive probands and P4—a family member. Dashed line represents the calculated mean audiogram.

Relevant audiological data were only available for Slovak hearing impaired subjects (n = 6) from five families. The age of these patients ranged between 6–23 years. All of them suffered from prelingual non-progressive bilateral moderate to profound sensorineural hearing loss (Fig 3), with a downsloping audiometric curve in most of the affected individuals. The hearing loss was partially compensated by conventional hearing aids in all but one subject with profound hearing loss. None of the tested cochlear implant users carried mutation in the *MARVELD2* gene.

In the Hungarian subgroup, all homozygous probands had bilateral severe to profound deafness with minimal residual hearing. The hearing loss onset was also in the prelingual period (diagnosed between 0–12 years).

## Discussion

### Prevalence and epidemiology of *MARVELD2* mutations

To date, only eleven families of Pakistani, three of Roma and one of Iranian origin with *MARVELD2*-associated deafness are known in the existing literature [3, 4, 7, 10, 9]. The most frequent mutation is the c.1331+2T>C, identified in nine families [4, 7, 10]. In our study, this mutation was found in homozygous form in twelve additional Roma families originating from two Central European countries (Slovakia and Hungary). The prevalence of *MARVELD2* related hearing loss was 3.5% in the group of deaf Slovak Roma individuals and 8.23% in deaf Hungarian Roma individuals. In contrast to our initial expectations, these prevalences, particularly in Slovakia, are lower than previously reported for the Roma population in the Czech Republic (15.8%) [10]. The difference could be explained by the small sample size of the Czech study (3 positive of 19 tested families) [10], or by the effect of genetic drift between populations. In Pakistani population, the prevalence of *MARVELD2* deafness in the hearing impaired subjects has been estimated at 1.30% [7, 30], which more closely resembles the data acquired in Slovakia.

When we look at the allele frequency of the c.1331+2T>C mutation, we can see a similar contrast between the groups of Slovak (3.85%) and Hungarian (10.0%) deaf Romanies. However, a 4.5 times higher difference in opposite direction was detected in the carrier rate of the *MARVELD2* mutation between control groups of healthy Slovak 4.5% (1:23) and Hungarian 1% (1:100) Roma individuals. The carrier rate in the Czech Romanies has been established at 2% (1:48) [10].

It is difficult to determine the exact prevalence of the c.1331+2T>C homozygotes in Slovakia due to missing data describing the number of deaf Romanies in the country. There are estimates that approximately 10,200 individuals of the total population in Slovakia counting about 5.5 million are bilaterally deaf [31]. According to data from Universal Newborn Hearing Screening Program gathered between 2009 and 2011, the incidence of congenital deafness in Slovakia is 1.20 per 1000 newborns (unpublished data). Moreover, it is believed a further 2–3 cases per 1000 newborns have congenital hearing loss to a milder degree [32]. Furthermore, if we consider that the Roma population in Slovakia represents about 7.45% of the total population [33] and the prevalence of c.1331+2T>C homozygotes in *GJB2* negative hearing impaired Romanies was shown to be 3.5%, we may estimate that *MARVELD2* related deafness in Slovakia may affect up to approximately 80 families. This calculation does not take into account the inbreeding rate, which in Slovakia is the highest recorded for a European Roma population [15]. Thus, the actual prevalence may be even higher and further screening in Roma subpopulations particularly in Eastern Slovakia will be required. Exact input data for Hungary and Czech Republic are not available, although similar numbers of patients (adjusted to the country populations) could be expected.

The other five pathogenic mutations of the *MARVELD2* gene, (c.1183-1G>A, c.1498C>T, c.1331+1G>A and c.1331+2delTGAG), to date only found in Pakistani [4, 7] and c.1543delA in Iranian population [9], were not detected by sequencing of the six coding regions in any of the 143 unrelated deaf Slovak Roma tested subjects. However, we have identified one variant with unknown pathogenicity (c.950G>A) leading to amino acid exchange (Arginine to Glutamine) at protein position 317. This variant was detected in five individuals in heterozygous form. It was not present in the 1000 Genomes project Phase 1 release (www.ncbi.nlm.nih.gov/variaton/tools/1000genomes/) [accessed 20 April 2014], but was detected in the NHLBI GO Exome Sequencing Project in one of 6502 individuals in heterozygous form (http://evs.gs.washington.edu/EVS/) [accessed 20 April 2014]. The structure-function relationship or clinical effect of the c.950G>A substitution are not yet known. The *In silico* programs PolyPhen2, SIFT and MutationTaster predicted c.950G>A to be probably pathogenic, with possible impact on protein features or splicing of mRNA. Further studies are required to evaluate the impact of this unknown variant.

By analyzing the c.1331+2T>C mutation in a Non-Roma (Caucasian) population (in a control group of 375 hearing impaired Slovak Caucasians), we have confirmed previous findings that occurrence of this mutation in Europe is probably restricted to the Roma population, and is still not known in Slavic Caucasian ethnicity [10].

### Haplotype analysis

**Fig 4:**
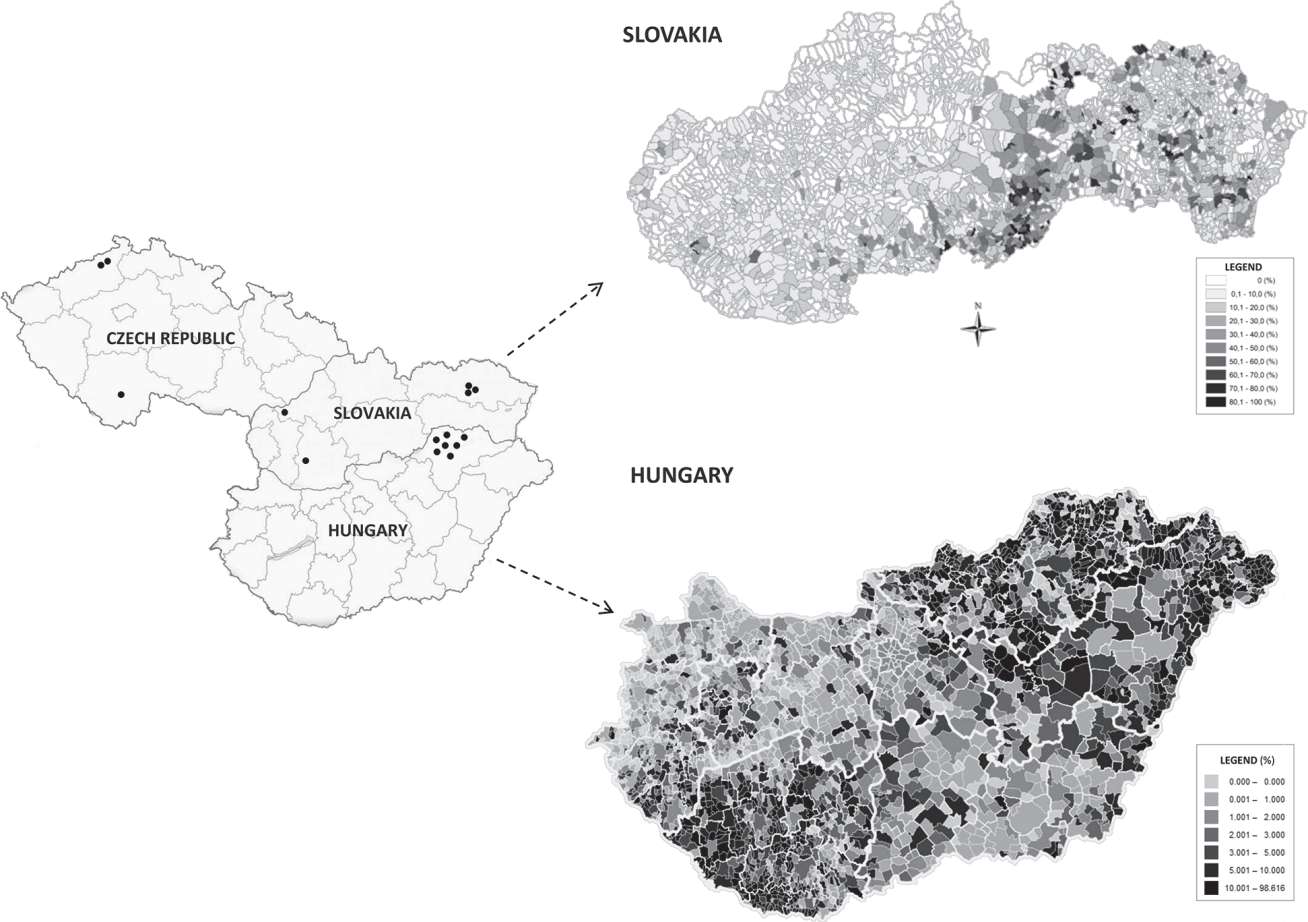
Distribution of the *MARVELD2* positive families in Slovakia and Hungary. Black points on the left hand side of the map represent c.1331+2T>C homozygous probands in the Czech Republic, Slovakia and Hungary. The maps of Slovakia and Hungary on right hand side show proportion rate of the Roma ethnicity at the municipality level [33, 34]. This data is not available for the Czech Republic.

Until now, *MARVELD2* homozygous patients in Europe have only been detected in Czech Republic [10], Slovakia and Northeastern Hungary (Fig 4). Their geographic distribution includes different regions, despite a presumed isolation of certain Roma subpopulations (clans) in these countries. Two Czech families are from the Northwest region (Ústí nad Labem) and one from South Bohemia (České Budějovice). Three Slovak families come from the Eastern (Prešov County) and two from the Western (Nitra and Trenčín County) part of the country. All Hungarian homozygous individuals originate from Northeastern Hungary (Borsod-Abaúj-Zemplén County). But this is actually due to the fact that DNA samples of hearing impaired Hungarian Roma patients were only available from this particular region.

When looking for the possible common ancestry, key historical events concerning the Central European Roma population must be taken into account. These 3 neighboring countries were inhabited by different Roma subpopulations. In the Czech Republic, nearly the entire original population of Bohemian Romanies was exterminated during the World War II holocaust in the Nazi concentration camps. Subsequently, according to the communist regime’s social engineering projects in former Czechoslovakia, Roma were moved from their original rural settlements in Slovakia (particularly from its eastern parts) to tenement blocks in Czech cities [35, 36]. Consequently, the vast majority of current Czech Romanies also originate from Slovakia. Dominant Roma subpopulations in the Slovak Republic include Romungro (Carpathian Roma) and Valachian (Olah) Roma. In Hungary, three major Roma groups, the Romungro (inhabiting most of the country), Valachian (in Northern Hungary) and Boyash (mostly in Southern Hungary) are recognized [37].

In the present study, we tested whether patients with the c.1331+2T>C mutation share a common ancestral haplotype. In seventeen Roma patients (5 Slovak, 5 Czech and 7 Hungarian), we genotyped 21 biallelic polymorphisms spanning approxximately 5.34 Mb around the mutation. Haplotype analysis revealed a common haplotype of 18 SNPs (approx. 3.9 Mb) which was present in the homozygous state in all Roma c.1331+2T>C patients but none of the 56 control Roma individuals. These data support the hypothesis of common ancestor for the c.1331+2T>C mutation in all analyzed Slovak, Czech and Hungarian Roma patients.

The origin of the founder Roma population is considered to be in Northwest India [12]. So far, *MARVELD2* positive deafness cases are only known from Pakistan, Iran and Czech Republic (Central Europe) [4, 7, 9, 10], not from India. Studies on two other diseases (Neuronal ceroid lipofuscinosis and Congenital myasthenia syndrome) have already revealed a presumed common ancestor for Roma and Pakistani populations [38, 39]. We therefore additionally analyzed two c.1331+2T>C Pakistani patients, whose haplotype was identical to European Roma patients and consists of fourteen SNPs (approx. 3.36Mb). This may indicate the same origin of c.1331+2T>C mutation in Roma and Pakistani patients. When we compared two Pakistani families, each containing different *MARVELD2* mutations, we only found 9 shared SNP markers (Fig 1).

### Hearing loss phenotype in c.1331+2T>C homozygous carriers

The phenotypes of six Slovak patients with *MARVELD2* related deafness (Fig 3) correspond to available literature data. Although most of the patients carrying biallelic c.1331+2T>C mutation suffer from profound deafness, hearing loss resulting from this mutation may range from moderately-severe to profound, with certain inter- and intrafamilial variability [3, 4, 7, 10]. The mean audiograms calculated from hearing thresholds in our six patients show a milder degree of hearing loss compared to all previous studies. Patients in whom serial audiograms were available, seemed to have a stable, non-progressive hearing loss. It may indicate that the severity of hearing impairment is fixed in the early prelingual period and the interindividual variability is not much affected by postnatal environmental factors. Unfortunately, exact audiological data (pure tone thresholds) from Hungarian patients were not available as only severity and age of onset of hearing loss were noted in the accessible clinical files. Based on these data, hearing loss associated with *MARVELD2* mutations is not known to develop in children older than 2–3 years, and may be considered congenital in vast majority of the cases.

## Conclusions

Our study provides the first record of *MARVELD2* related deafness in Slovakia and Hungary. It is also only the second study dealing with *MARVELD2* mutations in SNHL in Europe, as well as in the Roma population in general. We demonstrate distinct variability in prevalence of the c.1331+2T>C mutation in different Central European Roma populations, which is currently the only mutation of the tricellulin gene identified in this ethnicity. Haplotype analysis of the c.1331+2T>C mutation supports the hypothesis of a common ancestry for Slovak, Czech and Hungarian Roma patients, as well as a common origin of the mutation in Roma and Pakistani patients. From clinical point of view, we recommend testing for the c.1331+2T>C mutation in *GJB2* negative Roma cases with early-onset SNHL. However, in contrast to the previous study in Czech Roma, our results did not provide as much support to regard it as one of the most important genetic causes of nonsyndromic deafness in Romanies. Future studies focused on other Roma subpopulations throughout Europe (particularly in the Mediterranean area) may further illuminate the relative importance of *MARVELD2* gene as a deafness cause in Roma. According to recent data and our opinion, its testing is not necessary in the majority (Caucasian) population.

## References

[1] Schrijver. I Hereditary non-syndromic sensorineural hearing loss: transforming silence to sound. J Mol Diagn. 2004;6:275–84. 15507665 10.1016/S1525-1578(10)60522-3 PMC1867482

[2] Van Camp G, Smith RJ. Hereditary hearing loss homepage. Accessed 10 Sept 2014. Available: http://hereditaryhearingloss.org/

[3] Ramzan K, Shaikh RS, Ahmad J, Khan SN, Riazuddin S, Ahmed ZM, et al A new locus for nonsyndromic deafness DFNB49 maps to chromosome 5q12.3-q14.1. Hum Genet. 2005;116:17–22. 15538632 10.1007/s00439-004-1205-8

[4] Riazuddin S, Ahmed ZM, Fanning AS, Lagziel A, Kitajiri S, Ramzan K, et al Tricellulin is a tight-junction protein necessary for hearing. Am J Hum Genet. 2006;79:1040–51. 17186462 10.1086/510022 PMC1698716

[5] Ikenouchi J, Furuse M, Furuse K, Sasaki H, Tsukita S, Tsukita S. Tricellulin constitutes a novel barrier at tricellular contacts of epithelial cells. J Cell Biol. 2005;171:939–45. 16365161 10.1083/jcb.200510043 PMC2171318

[6] Mariano C, Silva SL, Pereira P, Fernandes A, Brites D, Brito MA. Evidence of tricellulin expression by immune cells, particularly microglia. Biochem Biophys Res Commun. 2011;409:799–802. 10.1016/j.bbrc.2011.05.093 21624353

[7] Chishti MS, Bhatti A, Tamim S, Lee K, McDonald ML, Leal SM, et al Splice-site mutations in the TRIC gene underlie autosomal recessive nonsyndromic hearing impairment in Pakistani families. J Hum Genet. 2008;53:101–5. 18084694 10.1007/s10038-007-0209-3 PMC2757049

[8] Nayak G, Lee SI, Yousaf R, Edelmann SE, Trincot C, Van Itallie CM, et al Tricellulin deficiency affects tight junction architecture and cochlear hair cells. J Clin Invest. 2013;123:4036–49. 10.1172/JCI69031 23979167 PMC3754262

[9] Babanejad M, Fattahi Z, Bazazzadegan N, Nishimura C, Meyer N, Nikzat N, et al A comprehensive study to determine heterogeneity of autosomal recessive nonsyndromic hearing loss in Iran. Am J Med Genet Part A. 2012;158A: 2485–92. 10.1002/ajmg.a.35572 22903915

[10] Šafka Brožková D, Laštůvková J, Štěpánková H, Krůtová M, Trková M, Myška P, et al DFNB49 is an important cause of non-syndromic deafness in Czech Roma patients but not in the general Czech population. Clin Genet. 2012;82:579–82. 10.1111/j.1399-0004.2011.01817.x 22097895

[11] Fraser AM. The gypsies: The Peoples of Europe 2nd ed. Oxford, UK & Cambridge, USA: Blackwell 1995.

[12] Moorjani P, Patterson N, Loh PR, Lipson M, Kisfali P, Melegh BI, et al Reconstructing Roma history from genome-wide data. PLoS One. 2013;8:e58633 10.1371/journal.pone.0058633 23516520 PMC3596272

[13] Mendizabal I, Lao O, Marigorta UM, Wollstein A, Gusmão L, Ferak V, et al Reconstructing the population history of European Romani from genome-wide data. Curr Biol. 2012;22:2342–9. 10.1016/j.cub.2012.10.039 23219723

[14] Kalaydjieva L, Gresham D, Calafell F. Genetic studies of the Roma (Gypsies): a review. BMC Med Genet. 2001;2:5 11299048 10.1186/1471-2350-2-5 PMC31389

[15] Ferak V, Sivakova D, Sieglova Z. [The Slovak gypsies (Romany)—a population with the highest coefficient of inbreeding in Europe]. Bratisl Lek Listy. 1987;87:168–75. 3580917

[16] Plásilová M, Stoilov I, Sarfarazi M, Kádasi L, Feráková E, Ferák V. Identification of a single ancestral CYP1B1 mutation in Slovak Gypsies (Roms) affected with primary congenital glaucoma. J Med Genet. 1999;36:290–4. 10227395 PMC1734351

[17] Gencik A. Epidemiology and genetics of primary congenital glaucoma in Slovakia. Description of a form of primary congenital glaucoma in gypsies with autosomal-recessive inheritance and complete penetrance. Dev Ophthalmol. 1989;16:76–115. 2676634

[18] Plásilová M, Feráková E, Kádasi L, Poláková H, Gerinec A, Ott J. et al Linkage of autosomal recessive primary congenital glaucoma to the GLC3A locus in Roms (Gypsies) from Slovakia. Hum Hered. 1998;48:30–3. 9463798 10.1159/000022778

[19] Kalanin J, Takarada Y, Kagawa S, Yamashita K, Ohtsuka N, Matsuoka A. Gypsy phenylketonuria: a point mutation of the phenylalanine hydroxylase gene in Gypsy families from Slovakia. Am J Med Genet. 1994;49:235–9. 8116675 10.1002/ajmg.1320490215

[20] Bôžiková A, Gabriková D, Sovičová A, Behulová R, Mačeková S, Boroňová I, et al The frequency of factor V Leiden and prothrombin G20210A mutations in Slovak and Roma (Gypsy) ethnic group of Eastern Slovakia. J Thromb Thrombolysis. 2012;34:406–9. 22562116 10.1007/s11239-012-0736-4

[21] Gabriková D, Bernasovská J, Mačeková S, Bôžiková A, Bernasovský I, Bališinová A, et al Unique frequencies of HFE gene variants in Roma/Gypsies. J Appl Genet. 2012;53:183–7. 10.1007/s13353-012-0088-y 22354660

[22] Schwabova J, Brozkova DS, Petrak B, Mojzisova M, Pavlickova K, Haberlova J, et al Homozygous EXOSC3 mutation c.92G—>C, p.G31A is a founder mutation causing severe pontocerebellar hypoplasia type 1 among the Czech Roma. J Neurogenet. 2013;27:163–9. 10.3109/01677063.2013.814651 23883322

[23] Baránková L, Sisková D, Hühne K, Vyhnálková E, Sakmaryová I, Bojar M, et al A 71-nucleotide deletion in the periaxin gene in a Romani patient with early-onset slowly progressive demyelinating CMT. Eur J Neurol. 2008;15:548–51. 10.1111/j.1468-1331.2008.02104.x 18410371

[24] Melegh B, Bene J, Mogyorósy G, Havasi V, Komlósi K, Pajor L, et al Phenotypic manifestations of the OCTN2 V295X mutation: sudden infant death and carnitine-responsive cardiomyopathy in Roma families. Am J Med Genet A. 2004;131:121–6. 15487009 10.1002/ajmg.a.30207

[25] Hunter M, Heyer E, Austerlitz F, Angelicheva D, Nedkova V, Briones P, et al The P28T mutation in the GALK1 gene accounts for galactokinase deficiency in Roma (Gypsy) patients across Europe. Pediatr Res. 2002;51:602–6. 11978884 10.1203/00006450-200205000-00010

[26] Minárik G, Ferák V, Feráková E, Ficek A, Poláková H, Kádasi L. High frequency of GJB2 mutation W24X among Slovak Romany (Gypsy) patients with non-syndromic hearing loss (NSHL). Gen Physiol Biophys. 2003;22:549–56. 15113126

[27] Seeman P, Malíková M, Rasková D, Bendová O, Groh D, Kubálková M, et al Spectrum and frequencies of mutations in the GJB2 (Cx26) gene among 156 Czech patients with pre-lingual deafness. Clin Genet. 2004;66:152–7. 15253766 10.1111/j.1399-0004.2004.00283.x

[28] Alvarez A, del Castillo I, Villamar M, Aguirre LA, González-Neira A, López-Nevot A, et al High prevalence of the W24X mutation in the gene encoding connexin-26 (GJB2) in Spanish Romani (gypsies) with autosomal recessive non-syndromic hearing loss. Am J Med Genet A. 2005;137A:255–8. 16088916 10.1002/ajmg.a.30884

[29] Bouwer S, Angelicheva D, Chandler D, Seeman P, Tournev I, Kalaydjieva L. Carrier rates of the ancestral Indian W24X mutation in GJB2 in the general Gypsy population and individual subisolates. Genet Test. 2007;11:455–8. 10.1089/gte.2007.0048 18294064

[30] Riazuddin S, Belyantseva IA, Giese AP, Lee K, Indzhykulian AA, Nandamuri SP, et al Alterations of the CIB2 calcium- and integrin-binding protein cause Usher syndrome type 1J and nonsyndromic deafness DFNB48. Nat Genet. 2012;44:1265–71. 10.1038/ng.2426 23023331 PMC3501259

[31] Kabatova Z, Profant M. [Deafness and cochlear implantation]. Via pract. 2007;4:76–8.

[32] National Institute of Deafness and other Communication Disorders. Accessed 20 May 2014. Available: www.nidcd.nih.gov/health/statistics/Pages/quick.aspx

[33] Musinka A, Skobla D, Hurrle J, Matlovicova K, Kling J. Atlas of Roma communities in Slovakia 2013 Bratislava: UNDP, 2014.

[34] Hungarian Central Statistical Office. Census 2011. Accessed 02 Dec. 2014. Available: http://www.ksh.hu/interaktiv/terkepek/mo/nemz_eng.html

[35] Koupilová I, Epstein H, Holcík J, Hajioff S, McKee M. Health needs of the Roma population in the Czech and Slovak Republics. Soc Sci Med. 2001;53:1191–204. 11556609 10.1016/s0277-9536(00)00419-6

[36] Lassuthova P, Sišková D, Haberlová J, Sakmaryová I, Filouš A, Seeman P. Congenital cataract, facial dysmorphism and demyelinating neuropathy (CCFDN) in 10 Czech gypsy children—frequent and underestimated cause of disability among Czech gypsies. Orphanet J Rare Dis. 2014;9:46 10.1186/1750-1172-9-46 24690360 PMC3976362

[37] Juhász E1, Béres J, Kanizsai S, Nagy K. The Consequence of a Founder Effect: CCR5-32, CCR2-64I and SDF1-3'A Polymorphism in Vlach Gypsy Population in Hungary. Pathol Oncol Res. 2012;18:177–82. 10.1007/s12253-011-9425-4 21667221

[38] Sharp JD, Wheeler RB, Parker KA, Gardiner RM, Williams RE, Mole SE. Spectrum of CLN6 mutations in variant late infantile neuronal ceroid lipofuscinosis. Hum Mutat. 2003;22:35–42. 12815591 10.1002/humu.10227

[39] Morar B, Gresham D, Angelicheva D, Tournev I, Gooding R, Guergueltcheva V, et al Mutation history of the roma/gypsies. Am J Hum Genet. 2004;75:596–609. 15322984 10.1086/424759 PMC1182047

